# The Structure of Ethylbenzene, Styrene and Phenylacetylene Determined by Total Neutron Scattering

**DOI:** 10.1002/cphc.201700393

**Published:** 2017-08-16

**Authors:** Joanna Szala‐Bilnik, Marta Falkowska, Daniel T. Bowron, Christopher Hardacre, Tristan G. A. Youngs

**Affiliations:** ^1^ School of Chemical Engineering and Analytical Science The University of Manchester Manchester M13 9PL UK; ^2^ STFC ISIS Facility Rutherford Appleton Laboratory Chilton Didcot Oxon OX11 0QX UK; ^3^ School of Chemistry and Chemical Engineering Queen's University Belfast Belfast BT9 5AG UK

**Keywords:** aliphatic chains, aromatic liquids, coordination sphere, liquid structure, neutron diffraction

## Abstract

Organic solvents such as phenylacetylene, styrene and ethylbenzene are widely used in industrial processes, especially in the production of rubber or thermoplastics. Despite their important applications detailed knowledge about their structure is limited. In this paper the structures of these three aromatic solvents were investigated using neutron diffraction. The results show that many of their structural characteristics are similar, although the structure of phenylacetylene is more ordered and has a smaller solvation sphere than either ethylbenzene or styrene. Two regions within the first coordination sphere, in which the surrounding molecules show different preferable orientations with respect to the central molecule, were found for each liquid. Additionally, the localisation of the aliphatic chains reveals that they tend to favour closer interactions with each other than to the aromatic rings of the adjacent molecules.

## 1 Introduction

Styrene is an important chemical that is critical to the production of many widely used polymers, such as polystyrene and synthetic rubbers. These materials have major value and find everyday use in objects such as films that are used for food containment, or in blocks of thermal insulation used in building construction. More highly optimized applications are also prevalent, such as its use in the production of rubbers used for vehicle tyres that are tailored to deliver enhanced performance and fuel efficiency. As a valuable source material, styrene itself is produced from ethylbenzene, usually by dehydrogenation using steam over an iron oxide catalyst,^1^, ^2^, ^3^ or equally can be produced by the reduction of phenylacetylene using hydrogen gas over a Lindlar catalyst.^4^ In the production of polymers, the purity of the source material is very important as contaminants can poison the polymerisation catalysts.^5^, ^6^ For styrene feedstock, particular care needs to be taken over the presence of residual ethylbenzene or phenylacetylene. Herein, we investigate the details of the intermolecular structure of these three closely related liquid reagents in their pure forms, with the aim of improving our understanding of how the nature the aliphatic side chain affects their molecular packing and potential reactivity.

The interactions between the aromatic ring and various species have drawn attention due to their importance in many systems. These include cation–π interactions which are amongst the strongest non‐covalent interaction and, in many cases, are stronger than hydrogen bonds and van der Waals interactions.^7^, ^8^ They are frequently encountered in nature and play a key role in biological systems such as cation complexes with proteins, chemical catalysis and solid‐state physics^7^, ^9^, ^10^ as well as in soil chemistry.^8^ In addition, OH–π interactions are thought to be important in determining protein structures and other biological assemblies and processes.^11^, ^12^, ^13^, ^14^ CH–π stacking between sugar and aromatic residues is universally used by lectins to interact with carbohydrates and is also involved in the binding of proteins to the sugar moiety of glycolipids.^15^, ^16^, ^17^, ^18^, ^19^, ^20^, ^21^, ^22^, ^23^ Moreover, π–π stacking interactions are common and these interactions between the aromatic residues also can stabilize protein structures.^24^ π–π mediated vertical base stacking also significantly contributes to the stability of the DNA double helix.^25^ The importance of these interactions illustrate the need to understand π–π bonding in detail. In this regard, benzene has been studied extensively using both experimental^26^, ^27^, ^28^, ^29^, ^30^ and theoretical^31^, ^32^, ^33^, ^34^, ^35^ methods and it has been shown to have a T‐shape perpendicular and parallel configurations which are almost isoenergetic. Although the T‐shape configuration is now viewed as the global energy minimum, the Y‐shape configuration is also found close to the global energy minimum.^31^ In the case of toluene, the increased dispersion interactions stabilize the parallel geometry^36^, ^37^ and the molecular dipole moment favours a stagger (anti‐parallel) arrangement of the methyl groups over the eclipsed geometry.^38^ Simulations of liquid benzene using classical atom—centred force fields have shown random orientations or a slight preference for perpendicular arrangements of nearest neighbor molecules,^39^, ^40^, ^41^ but simulations using partial charges for benzene yield a higher preferences for perpendicular arrangements of nearest neighbor molecules.^42^, ^43^ Liquid toluene has received less attention although it is viewed as a better model for π–π interaction in proteins.^44^ However, recent simulations indicate a prevalence of parallel stacking of the aromatic planes, with the staggered disposition of ‐CH_3_ groups.^38^, ^45^, ^46^ Aiming to resolve key aspects of conflicting pictures arising from the simulation and earlier experimental work, neutron diffraction studies of benzene and toluene have subsequently been reported by Headen et al.^47^ and Falkowska et al..^48^ Headen et al.^47^ analysed the structure of benzene and toluene and found that benzene is the more structured of the two liquids. Moreover, the multidimensional analysis showed that the local orientation order in these liquids is much more complex. At short separations the most favoured nearest neighbour geometry is parallel. At longer distances the perpendicular Y‐shape arrangement was preferable. Falkowska et al.^48^ confirmed these observations. A MM/QM study has shown that for benzene at short distances, molecules have parallel organisation, and at longer distances the preferred molecular configuration is for perpendicular organisation. The most stable configuration in the latter is a T‐shape arrangement,^44^ with the planes of the rings perpendicular to each other. This perpendicular arrangement is in contrast to the studies of Headen et al.^47^ and Falkowska et al.^48^ As part of an ongoing study to understand the liquid structure of cyclic hydrocarbons, this paper presents a neutron scattering study of the liquid structure of phenylacetylene, styrene and ethylbenzene as pure liquids to probe the influence of the aliphatic chain on the π–π interactions. Previous works have examined the structure of liquid benzene and toluene, which differ by a methyl group, and in these cases significant differences in structure were revealed. Relatively few studies have been reported about phenylacetylene liquid structure and they have revealed that the most stable molecular configurations are the anti‐parallel π‐stacked structure.^49^−^51^ In contrast, no reports, to date, have been published on the liquid structure of ethylbenzene or styrene.

## 2 Results and Discussion

**Figure 1:**
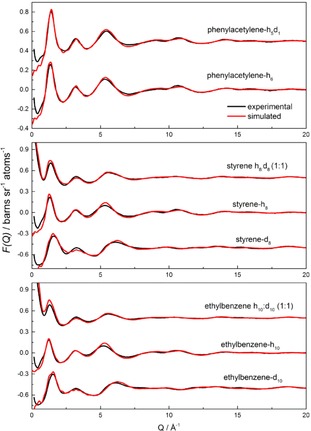
Experimental (black line) and EPSR‐fitted (red line) interference differential cross‐sections as a function of *Q* for phenylacetylene, styrene and ethylbenzene.

**Figure 2:**
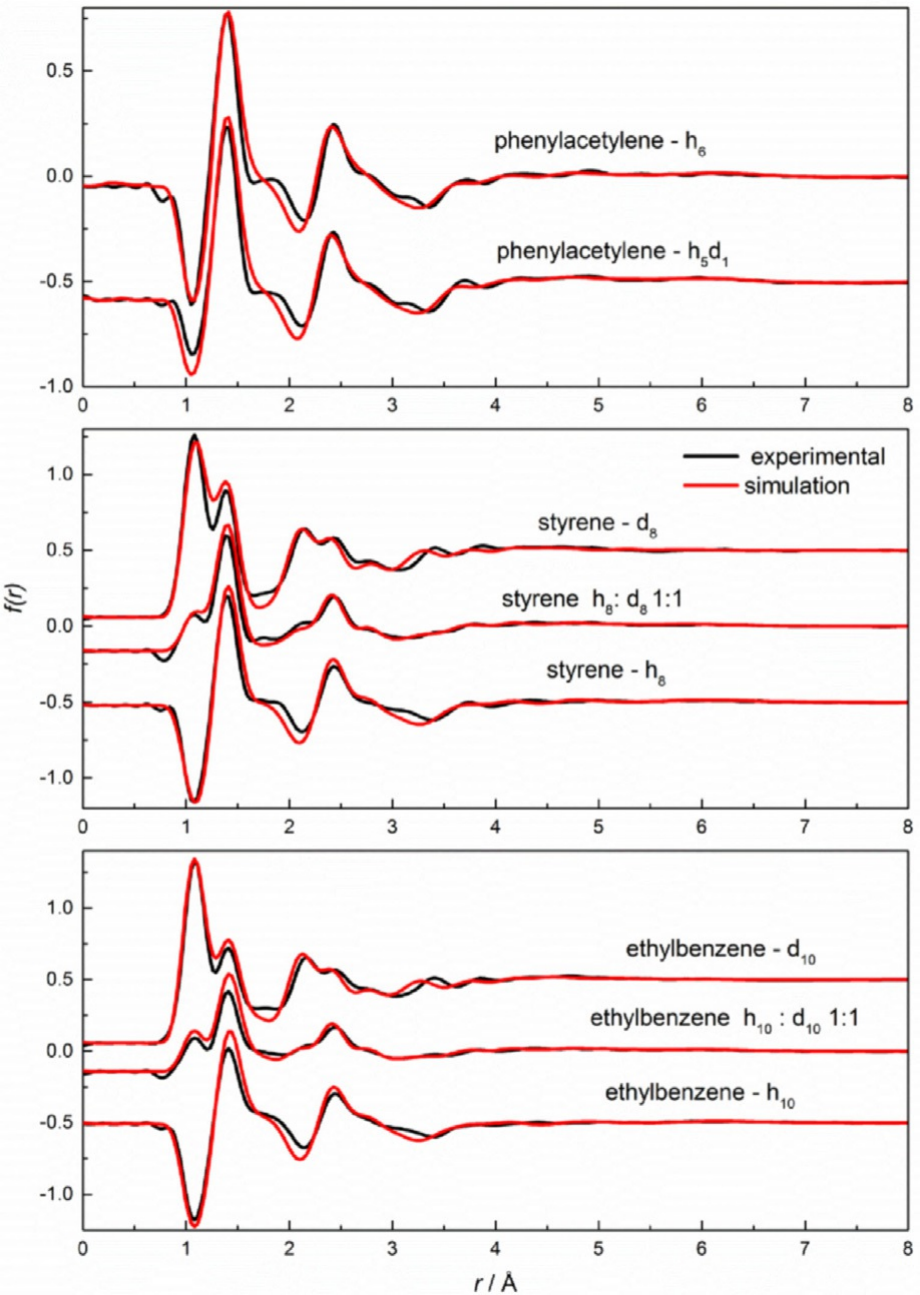
Experimental (black line) and EPSR‐fitted (red line) Fourier transform of F(Q) as a function of distance *r* for phenylacetylene, styrene and ethylbenzene.

Neutron diffraction data and Empirical Potential Structure Refinement (EPSR) fits for phenylacetylene, styrene and ethylbenzene are shown in Figure 1. Good agreement between experimental and simulated data was found in all cases. A small residual disagreement at lower *Q* values was found and attributed to errors in the subtraction of inelasticity effects from the data. The better fitting for the deuteriated samples is consistent with this proposal, as these samples are less prone to inelasticity effects. Figure 2 presents the Fourier transform of the F(*Q*) data as a function of distance, *r*. The agreement between the experimental and simulated data is also good.

**Figure 3:**
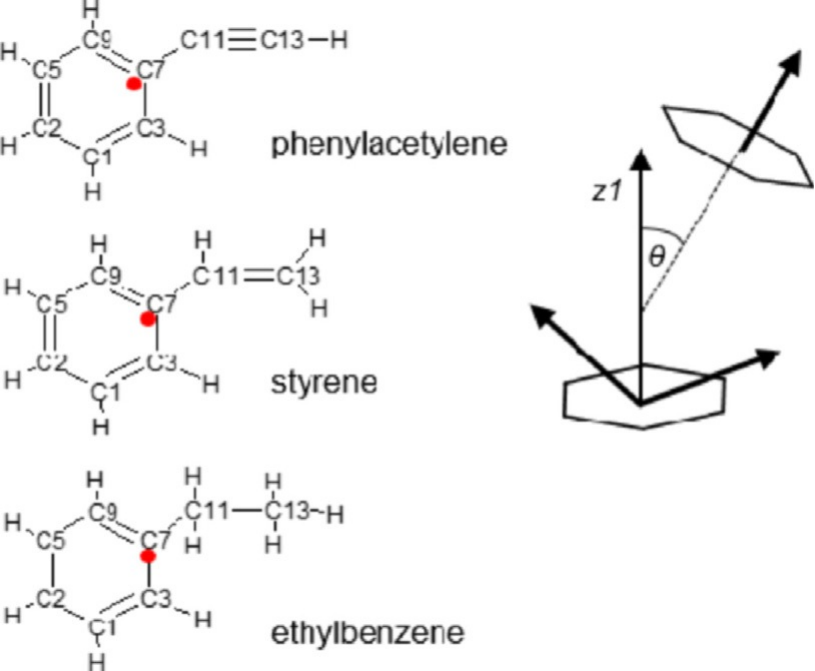
Schematic showing the notation used for the atom assignments and the definition of the angle *θ* between molecules. The red dots show the centre of the geometry (CoG) of each molecule. All CoG are in the plane of the ring.

The data obtained from the EPSR modelling was used to calculate the radial distribution functions (RDFs), angular radial distribution functions (ARDFs) and spatial probability of density functions (SPDFs). Figure 3 shows the notation used in the paper for the identification of the atoms and also the definition of the angle *θ* between the *z* axis of the central and surrounding molecules used in the calculation of the angular radial distribution function.

Figure S1 in the Supporting Information shows the RDFs between the centres of geometry (main) and the coordination number (inset) as the function of distance for phenylacetylene, styrene and ethylbenzene. In each case small differences are found, for example, in the positions of the first maximum, first minimum, second maximum and the coordination numbers for the first shell for each liquid (Table 1). For each liquid, two well defined solvation shells were found. For phenylacetylene, the first coordination shell maximum was found at 5.95 Å. For styrene and ethylbenzene, slightly lower maxima are found at 6.25 Å and at 6.35 Å, respectively.

**Table 1:** Positions of first maximum, minimum, second maximum and the coordination number of the first shell for phenylacetylene, styrene and ethylbenzene. The errors are ±0.1 Å and ±0.4 for the distances and coordination numbers, respectively.

Compound	1^st^ maximum [Å]	1^st^ minimum [Å]	2^nd^ maximum [Å]	1^st^ shell coordination number
phenylacetylene	5.95	8.15	10.25	12.0
styrene	6.25	8.55	10.85	13.3
ethylbenzene	6.35	8.65	11.05	12.9

**Figure 4:**
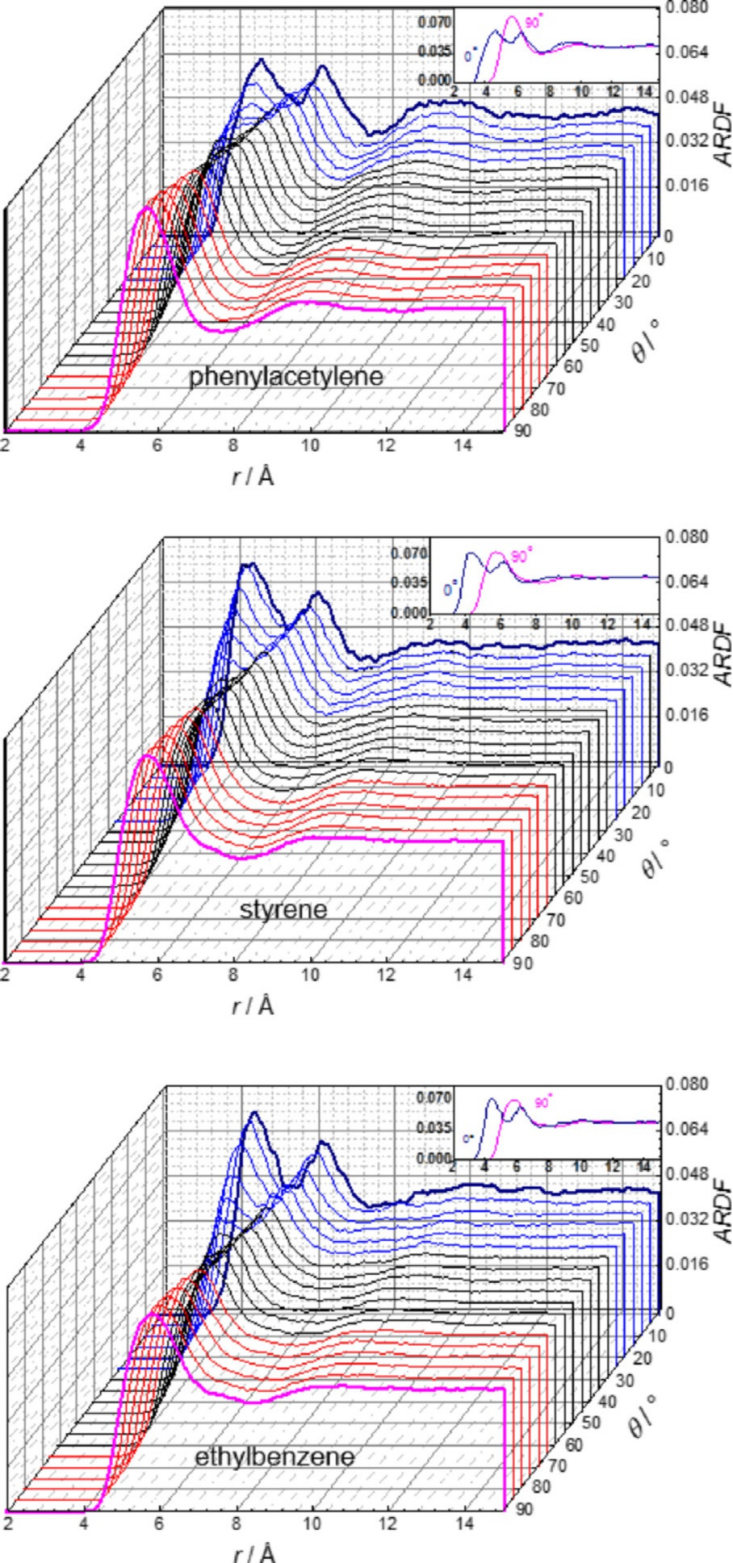
Angular radial distribution function for phenylacetylene (top), styrene (middle) and ethylbenzene (bottom) calculated as a function of the angle between the *z* axes of the central and surrounding molecules (0°<*θ*<90°), as shown in Figure 3. The blue lines represent angles smaller than 30° with thick dark blue line corresponding to 0°. The red lines represent angles larger than 60° with 90° denoted by the thick pink line. Data for the two extreme angles (in pink and dark blue) are also presented as an inset for direct comparison.

**Figure 5:**
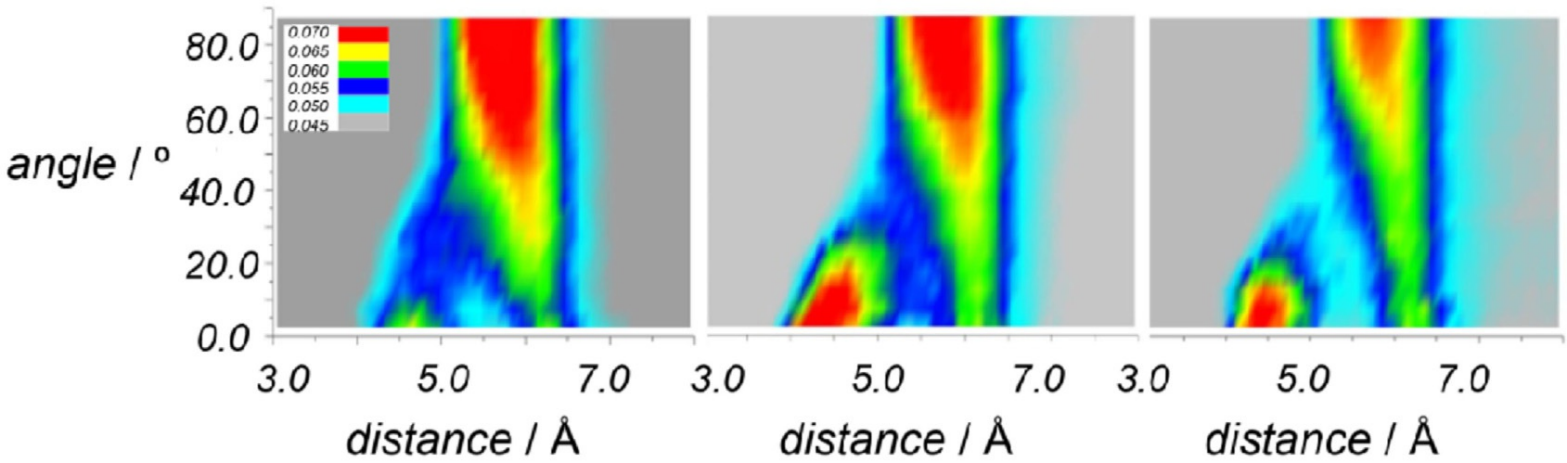
The overhead projection of the 3D plot (Figure 4) for phenylacetylene (left), styrene (middle) and ethylbenzene (right) calculated as a function of the angle between the *z* axes of the central and surrounding molecules (0°<*θ*<90°), as shown in Figure 3.

The second maxima were found at 10.25, 10.85 and 11.05 Å, respectively. Table 3 shows the first shell coordination numbers are between 12.0 and 13.3 and follow the trend styrene>ethylbenzene>phenylacetylene. The shorter first shell coordination distance suggests that the structure of phenylacetylene is more tightly packed than the other two solvents, which could be due to the smaller size of the aliphatic chain or to the presence of stronger local ordering. To analyse the orientation of the molecules in the coordination shells around the central molecule angular radial distribution functions (ARDF), that is, the RDF plotted as a function of the angle *θ* between the *z* axes of the central molecule and surrounding molecules, were calculated and are presented in Figure 4, Figure 5 and Figure S2 in the Supporting Information. The positions of the maxima are summarised in Table 2.

**Table 2:** Positions of first maximum, minimum, second maximum of the angular radial distribution function of 0° and for of 90° phenylacetylene, styrene and ethylbenzene. The error is 0.1 Å.

Compound	1^st^ maximum of 0° [Å]	2^nd^ maximum of 0° [Å]	1^st^ maximum of 90° [Å]
phenylacetylene	4.65	6.25	5.65
styrene	4.15	6.15	5.75
ethylbenzene	4.45	6.15	5.85

The 0° ARDF, corresponding to parallel orientation of the molecules, has two maxima for each system, at 4.5 Å–4.7 Å and at ≈6.5 Å. The height of the first maximum is similar for styrene and ethylbenzene with a peak value of 0.74, but lower by ≈12 % for phenylacetylene. The second maximum has the same position and magnitude for all solvents. The 90° ARDF, assigned to a perpendicular orientation, has only one maximum in each case at 5.65 Å for phenylacetylene, at 5.75 Å for styrene and at 5.85 Å for ethylbenzene. The maximum is the highest for phenylacetylene and the lowest for ethylbenzene. Only the ARDF of phenylacetylene has a well‐defined minimum and second maximum around 9.5 Å.

The presented ARDFs reveal that, at short distances, the molecules prefer to be in a parallel orientation. In contrast, the molecules adopting the perpendicular arrangement were found at longer separations, but parallel orientation is also possible at these distances. At the longer distances, >5 Å, the intensity of the ARDFs for phenylacetylene are higher than for the other solvents showing that the structure of phenylacetylene is more structured. The more clearly defined positions of the minimum and second maximum of 90° ARDF is also consistent with this view. For all the systems, the position of the second maximum of 0° ARDF is found at longer separations than found for the maximum of the 90° ARDF which is consistent with a second layer of the parallel molecules being present. At distances >7.5 Å there is no strong preference for any specific orientation. The populations of the parallel and perpendicular molecules for each liquid were calculated and the results are summarised in Table 3. The short distance cut‐off corresponds to the first minimum of the 0° ARDFs and long distance cut‐off with second minimum of the 0° ARDFs. The minimum of the 90° ARDFs was found at similar distance to minimum of 0° ARDF and, therefore, this value was used as the minimum of the 90° ARDF. No significant difference was found between molecules studied. At shorter distances from the central molecule, more molecules were found to be parallel than at longer separations, but in all cases the number of perpendicular orientations is far higher than the parallel orientations. However, the number of all specifically oriented molecules accounts for only a small portion (≈21 %) of the total number of molecules in the first coordination sphere. It is important to emphasise that, although the specific configurations may be important for the chemical behaviour, most of the molecules have generally disordered intermolecular interactions.

**Table 3:** The populations of parallel and perpendicular molecules of all molecules present in the specific range. For each liquid, such as phenylacetylene, styrene and ethylbenzene, the shorter (0–5.65 Å, 0–5.35 Å and 0–5.55 Å), and longer distances (5.65–7.45 Å, 5.35–7.65 Å and 5.55–7.45 Å) are determined from the corresponding ARDFs. The errors are ≈0.5 %.

Compound	Parallel [%]		Perpendicular [%]	
	Short distance	Long distance	Short distance	Long distance
phenylacetylene	1.0	0.7	15.8	17.8
styrene	1.5	0.7	12.8	20.8
ethylbenzene	1.2	0.7	14.7	17.9

**Figure 6:**
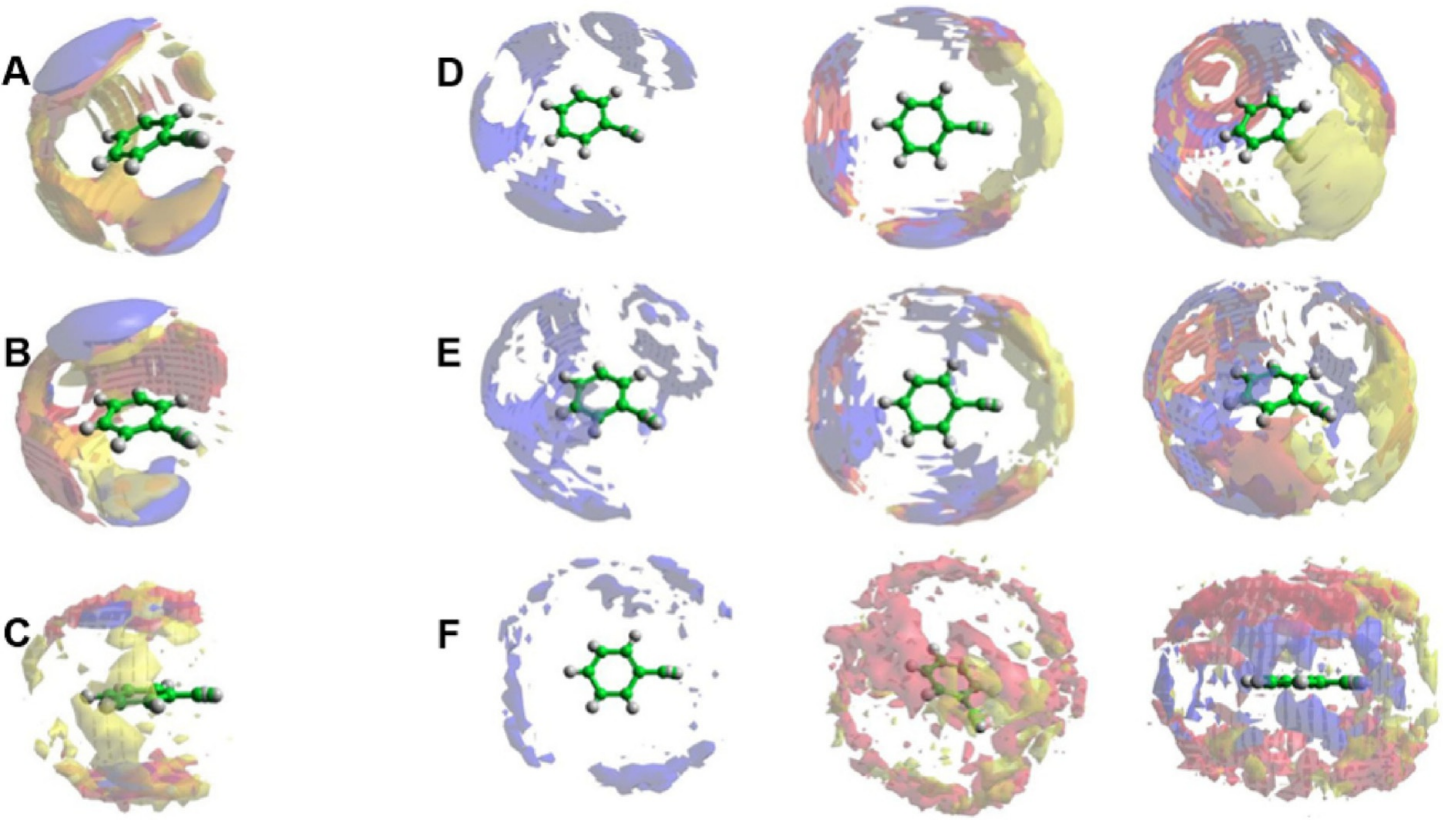
Spatial probability density functions for liquid phenylacetylene calculated within two distances ranges determinate from ARDF, that is, 0–5.65 Å and 5.65–7.45 Å from the central molecule. The function represents 20 % of A) all molecules, B) perpendicular molecules only (*θ*=90±10°) and C) parallel molecules only (*θ*=0±10°) with respect to the central molecule found within *r*=0–5.65 Å. Additionally, functions representing the top 10 % of D) all molecules, E) perpendicular molecules only (*θ*=90±10°) and F) parallel molecules only (*θ*=0±10°) with respect to the central molecule found within *r*=5.65–7.45 Å. Three different perspectives with only centre of geometry (blue) and centre of geometry and C11 (red) and C13 atoms (yellow surface) are shown for D, E and F to illustrate the structures.

To study the three‐dimensional organisation of molecules, the spatial probability density functions were calculated for each system. In this approach, the position of surrounding molecules or chosen atoms is then binned on a three‐dimensional grid according to their position from the central molecule. The number of molecules/atoms found in a given ‘bin’ is indicative of the ‘popularity’ of that position and so, when averaged over all molecules/atoms and many frames, these ‘spatial probability densities’ offer a snapshot of the preferred positions of one species relative to another. Plotting a surface which encompasses all positions above a certain threshold (a useful measure is typically the bulk number density of the molecule/atom type) visually illustrates probable average positions of the molecules/atoms in 3D space. Such functions are shown in Figure 6 for phenylacetylene. These functions look similar for styrene and ethylbenzene and are presented in Figure S3 and Figure S4, respectively.

The analysis of the nearest molecules from the central molecule shows that at short distances the centres of geometry of the surrounding molecules are localised above and below the aromatic ring, and includes both parallel and perpendicular orientation. It is observed that the centres of geometry of the surrounding molecules are displaced away from the aliphatic group towards the centre of the ring of central molecule. The carbon atoms of aliphatic chains are placed equatorially in defined areas around the central molecule and also above and below the ring. For the molecules in perpendicular orientations similar observations are found. However, in the parallel orientation the carbon atoms of the chain (Figure 6 C) are found above and below the ring, but without a defined orientation. Again the aliphatic group is displaced away from the aliphatic chain of the central molecule. The positions of the atoms around central molecule suggest that the parallel orientation at short distances is preferable, but it is not a parallel stacked arrangement. The distributions of the alkyl chain of surrounding molecules away from the alkyl chain of the central molecule are consistent with an *anti*‐parallel arrangement or displaced parallel arrangement for each system.

The analysis of the orientation of molecules at longer separations reveals that the most probable positions of the surrounding molecules are well‐defined around the equator of the central molecule. Such an organisation corresponds to a Y‐shape arrangement. Compared the position of the perpendicular molecules with the position of the aliphatic chain at short separation, an overlapping of the positions is noticeable. This suggests that the aliphatic chain attached to perpendicular molecules is placed between perpendicular and central molecule, towards to the aromatic ring. At longer separations the carbon atoms of the aliphatic chain were found mostly to be close to the aliphatic chain of the central molecule revealing an association where the aliphatic chains come together. If the molecules are parallel at long separations, they are localized equatorially and the aliphatic chains make halos above and below the ring.

**Figure 7:**
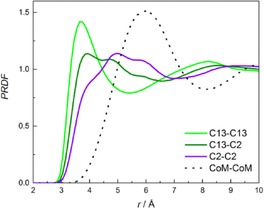
Site–site radial distribution functions for phenylacetylene. The PRDF between the carbon atoms of the aliphatic chains (C13–C13) are in green, carbon atom of the aliphatic chain–carbon atom of the ring (C13–C2) in dark green, carbon atoms of the ring (C2–C2) in violet and centre of geometry–centre of geometry (CoG–CoG) in dotted black line.

Figure 7 and Figure S5 show site‐site radial distribution functions for phenylacetylene and the RDFs for styrene and ethylbenzene are shown in Figure S6 and Figure S7, respectively. For all systems, the highest maximum and at shortest distance was found for C13–C13 (carbon atom of the aliphatic chain) PRDF revealing that the probability of finding the aliphatic chains close to each other is higher than to the ring. This is consistent with the proposal that the aliphatic chains interact. However, a smaller maximum at a longer distance for C13–C2 (carbon atom of the ring) PRDF shows that the aliphatic chain can also be found close to the ring of central molecule. The maximum of C2–C2 (carbon atoms of the ring) was found at longer distances of ≈4.9 Å, and the maximum of centre of geometry–centre of geometry (CoG–CoG) PRDF at 5.95 Å confirming that the central molecule is predominantly surrounded by the aliphatic chain. The radial distribution functions are similar for all three molecules; however, the positions of the maxima are shifted to longer distances for styrene and even longer distances for ethylbenzene as can be seen in Figure S8. This again is consistent with the proposal that the structure of the phenylacetylene is best defined.

Previous studies concerning the structure of ethylbenzene, styrene and phenylacetylene, are limited. Ab initio studies of the structure of phenylacetylene showed that the most stable (global minimum) is the anti‐parallel arrangement; however, other parallel orientations were also found to be highly probable.^49^, ^50^, ^51^ In the present experimental investigation, the most preferable orientation for the closest coordinating molecules were found to be in a parallel arrangement. The two structures for perpendicular molecules, in which two C−H⋅⋅⋅π hydrogen bonds between phenylacetylene molecules are present,^49^, ^50^ were also found as a local minimum, but the stabilization energy is less favourable than for parallel orientation. In our studies, there was no proof that would allow us to confirm or contradict the existence of these kinds of perpendicular arrangements for short separation.

Each of the studied molecules comprises an aromatic ring with a different aliphatic chain attached, and which could influence the structure of the liquids. The electrostatics based on Hunter‐Sanders π–π stacking model suggests that an electron donating substituent on one of the constituents and an electron withdrawing substituent on the other will lead to favourable π–π stacking.^52^, ^53^ In contrast, high‐level ab initio calculations indicates that any substitution, whether electron donating or electron withdrawing, will favour the formation of π–π stacking.^54^, ^55^ Substituent effects in the sandwich configuration of the benzene dimer^52^ are often rationalized in terms of a simple electrostatic model :^56^, ^57^ electron‐withdrawing substituents enhance the π‐stacking interaction by withdrawing π‐electron density from the substituted benzene, reducing the electrostatic repulsion with the other benzene. Electron‐donating substituents diminish π‐stacking interactions by the opposite mechanism. The aliphatic groups attached to aromatic ring in our studies belong to both electron‐withdrawing (ethynyl group) and donating substituents (vinyl and ‐C_2_H_5_ group); however, no strong and visible difference in the interaction between parallel solvent molecules was found. The contribution of the parallel molecules to the primary coordination sphere is also not significantly different between these three liquids. A comparison of the three molecules with previous studies for toluene and benzene^47^, ^48^ shows some common features. A recent previous study^48^ reveals that benzene and toluene shared common structural features in the liquid state, but that differences in the molecular organisation within the first solvation shell were affected by the presence of the methyl substituent. These results together with the findings from the present study, shows that the solvation spheres have similar sizes and coordination numbers for all five molecules. The smallest molecular separation occurs for benzene, this increases with increasing size of the chain (acetyl < methyl < vinyl < ethyl). For each of the molecules, two regions within the first coordination sphere, in which surrounding molecules show different behaviour in approaching to the central molecule, were found. At shorter distances, molecules prefer to be parallel and at longer separation the perpendicular orientation is preferred (although parallel molecules may still be present at these separations). It is important to emphasize that for benzene and toluene the maximum of the ARDF for the parallel orientation was found at 4 Å, while for the other molecules studied in this study the maxima occur at 4.5 Å. The maximum of ARDF of perpendicular arrangement in all cases was found at 6.0 Å. However, comparing quantitatively the number of parallel and perpendicular molecules with respect to all the molecules, for benzene at short separations more molecules are parallel than perpendicular. This is in the contrast with the four other molecules studied. In all cases, when the molecules are parallel, the parallel‐displaced arrangement is observed, but for benzene and toluene^47^, ^48^ this is much more obvious than for the molecules examined, herein. At long distances, for all solvents, the population of perpendicular molecules is dominant compared to parallel, and the Y‐shape arrangement was found for all investigated systems.

There is a strong dependence of the stacking arrangements with respect to the size of the chain on the aromatic ring. For toluene,^48^ the intensity and position of maximum of C chain—C chain PRDF and C chain—C ring PRDF are similar, around 4.2 Å, but for phenylacetylene, styrene and ethylbenzene, the maximum of C chain—C chain PRDF is higher than C chain—C ring PRDF. The position of maximum chain‐chain increases from 3.9 to 4.2 Å from phenylacetylene to ethylbenzene. This shows, that for toluene both interactions are equally probable, thus for three other solvents, the stacking of chains is more preferred.

## 3 Conclusions

The structures of phenylacetylene, styrene and ethylbenzene have been studied by neutron diffraction measurements to establish the influence of the saturation of the aliphatic chain attached to aromatic ring on the local ordering. Despite the small difference in the functionality of the aromatic molecules, only small differences were found in the intermolecular interactions. The size of the coordination sphere and the number of molecules contained within this shell are similar, with a slightly lower occupancy for phenylacetylene. The most clearly defined short range intermolecular structure was found for phenylacetylene and this is mostly due to the smaller size of the side‐chain attached to aromatic ring. The analysis of the organisation of the molecules in the shell reveals two regions, in which the surrounding molecules show different behaviour in their approach to the central molecule. At short separation molecules prefer to be parallel, and at longer separations the perpendicular arrangement is dominant, but parallel orientations also exist. An examination of the aliphatic chain interactions also demonstrated that there is a preference for chain‐chain proximity.

## Experimental Section

All neutron diffraction data were collected using the Small Angle Neutron Diffractometer for Amorphous and Liquid Samples (SANDALS) at the ISIS pulsed neutron and muon source at the Rutherford Appleton Laboratory, UK.^58^

For ethylbenzene and styrene, scattering data on the fully protiated and fully deuteriated compounds and their equimolar mixture were collected to take full advantage of the isotopic contrast between hydrogen and deuterium. For the phenylacetylene, scattering data on the fully protiated and single deuteriated on the acetyl chain compounds were collected. All chemicals were purchased from Sigma–Aldrich and used without further purification. The liquids were loaded into null‐scattering Ti_0.68_Zr_0.32_ flat plate containers of internal dimensions 35×40×1 mm and wall thickness of 1 mm. During the measurements, the samples were maintained at a temperature of 298 K. Additional measurements were made on each of empty sample cells, the empty diffractometer and a 3.0 mm thick vanadium standard sample. Data were corrected for multiple scattering and absorption, and normalized to the incoherent scattering of vanadium using the Gudrun software.^59^ Residual inelasticity effects arising from the presence of hydrogen were removed using iterative procedures.^59^

The corrected neutron diffraction data were analysed using the Empirical Potential Structure Refinement (EPSR) method, which allows the construction of three dimensional atomistic structural models of liquids or disordered samples that are consistent with experimental scattering data.^60^ After obtaining the agreement between the simulated and experimental data, the structural properties such as radial distribution functions, angular distribution functions and spatial distribution functions were calculated. For each liquid, EPSR analysis was initialized from an equilibrated Monte Carlo simulation at 298 K containing 400 molecules in a cubic box at the atomic densities presented in Table 4. Reference potential parameters for each pure liquid were taken from the OPLS‐AA force field,^61^ Table 5. Structural properties were calculated using a combination of built‐in routines in EPSR^60^ and custom analysis codes.^62^

**Table 4:** Simulation box sizes and densities.

Compound	Density [atoms Å^−3^]	Box length [Å]
phenylacetylene	0.0768	41.78
styrene	0.0841	42.38
ethylbenzene	0.0885	43.34

**Table 5:** Lennard–Jones potential and charges used in EPSR simulations for phenylacetylene, styrene and ethylbenzene. Atom types are applied as shown in Figure 1.

Species	Atom type	ϵ [kJ mol^−1^]	σ [Å]	q [e]
phenylacetylene	C1, C2, C3, C5, C9	0.29300	3.55	−0.115
	C7	0.31795	3.55	0.115
	C11	0.31795	3.55	0.115
	C13	0.31795	3.55	−0.115
	all H	0.12552	2.42	0.115
styrene	C1, C2, C3, C5, C9	0.29300	3.55	−0.115
	C7	0.31795	3.55	−0.115
	C11	0.31795	3.55	0.000
	C13	0.31795	3.55	−0.230
	all H	0.12552	2.42	0.115
ethylbenzene	C1, C2, C3, C5, C9	0.29300	3.55	−0.115
	C7	0.31795	3.55	−0.115
	C11	0.31795	3.55	−0.005
	C13	0.31795	3.55	−0.180
	ring H	0.12552	2.42	0.115
	aliphatic H	0.12552	2.50	0.060

## Conflict of interest

*The authors declare no conflict of interest*.

## Supporting information

As a service to our authors and readers, this journal provides supporting information supplied by the authors. Such materials are peer reviewed and may be re‐organized for online delivery, but are not copy‐edited or typeset. Technical support issues arising from supporting information (other than missing files) should be addressed to the authors.

Supplementary

## References

[1] T. Hirano , Appl. Catal. 1986, 26, 81–90.

[2] K. K. Kearby , “Catalytic Dehydrogenation” in *Catalysis, Vol. 3,* (Ed.: P H. Emmett), Reinhold, New York, **1955**, pp. 453–491.

[3] H. Lindlar , R. Dubuis , Org. Synth. 1966, 46, 89–90.

[4] Encyclopedia of Chemical technology, (Ed.: Kirk-Othmer), John Wiley & Sons, New York, 2008, pp. 1040.

[5] B. R. Maurer, M. Galobardes **1989**, *U.S. Patent No. 4*, 822, 936.

[6] T. Vergunst , F. Kapteijn , J. A. Moulijn , Ind. Eng. Chem. Res. 2001, 40, 2801–2809.

[7] J. C. Ma , D. A. Dougherty , Chem. Rev. 1997, 97, 1303–1324. 11851453 10.1021/cr9603744

[8] M. Keiluweit , M. Kleber , Environ. Sci. Technol. 2009, 43, 3421–3429. 19544834 10.1021/es8033044

[9] G. W. Gokel , L. J. Barbour , S. L. De Wall , E. S. Meadows , Coord. Chem. Rev. 2001, 222, 127–154.

[10] J. P. Gallivan , D. A. Dougherty , Proc. Natl. Acad. Sci. USA 1999, 96, 9459–9464. 10449714 10.1073/pnas.96.17.9459 PMC22230

[11] J. L. Atwood , F. Hamada , K. D. Robinson , G. W. Orr , R. L. Vincent , Nature 1991, 349, 683–684.

[12] G. V. Janjić , S. N. Malkov , M. V. Živković , S. D. Zarić , Phys. Chem. Chem. Phys. 2014, 16, 23549–23553. 25271703 10.1039/c4cp00929k

[13] A. J. Johnston , Y. Zhang , S. Busch , L. C. Pardo , S. Imberti , S. E. McLain , J. Phys. Chem. B 2015, 119, 5979–5987. 25893741 10.1021/acs.jpcb.5b02476

[14] J. M. Maier , P. Li , E. C. Vik , C. J. Yehl , S. M. Strickland , K. D. Shimizu , J. Am. Chem. Soc. 2017, 139, 6550–6553. 28463006 10.1021/jacs.7b02349

[15] M. Nishio , M. Hirota , Y. Umezawa , The CH/π Interaction. Evidence, Nature, and Consequences, Wiley-VCH Inc., New York 1998.

[16] N. K. Vyas , M. N. Vyas , F. A. Quiocho , Science 1988, 242, 1290–1295. 3057628 10.1126/science.3057628

[17] F. A. Quiocho , Annu. Rev. Biochem. 1986, 55, 287–315. 3527044 10.1146/annurev.bi.55.070186.001443

[18] M. Maresca , A. Derghal , C. Carravagna , S. Dudin , J. Fantini , Phys. Chem. Chem. Phys. 2008, 10, 2792–2800. 18464996 10.1039/b802594k

[19] V. Spiwok , P. Lipovova , T. Skalova , E. Buchtelova , J. Hasek , B. Kralova , Carbohydr. Res. 2004, 339, 2275–2280. 15337456 10.1016/j.carres.2004.06.016

[20] V. Spiwok , P. Lipovova , T. Skalova , E. Vondrackova , J. Dohnalek , J. Hasek , B. Kralova , J. Comput. Aided Mol. Des. 2005, 19, 887–901. 16607570 10.1007/s10822-005-9033-z

[21] W. R. Carroll , C. Zhao , M. D. Smith , P. J. Pellechia , K. D. Shimizu , Org. Lett. 2011, 13, 4320–4323. 21797218 10.1021/ol201657p

[22] J. Fantini , Cell. Mol. Life Sci. 2003, 60, 1027–1032. 12866532 10.1007/s00018-003-3003-1 PMC11138889

[23] J. Fantini , N. Garmy , N. Yahi , Biochemistry 2006, 45, 10957–10962. 16953581 10.1021/bi060762s

[24] S. K. Burley , G. A. Petsko , Science 1985, 229, 23–28. 3892686 10.1126/science.3892686

[25] R. Rein in I ntermolecular interactions: from diatomics to biopolymers,*ed. B. Pullman*, John Wiley & Sons, Chichester 1987, ch. 3, pp. 307–362.

[26] K. C. Janda , J. C. Hemminger , J. S. Winn , S. E. Novick , S. J. Harris , W. Klemperer , J. Chem. Phys. 1975, 63, 1419–1421.

[27] J. M. Steed , T. A. Dixon , W. Klemperer , J. Chem. Phys. 1979, 70, 4940–4946.

[28] K. S. Law , M. Schauer , E. R. Bernstein , J. Chem. Phys. 1984, 81, 4871–4882.

[29] V. Spirko , O. Engkvist , P. Soldan , H. L. Selzle , E. W. Schlag , P. Hobza , J. Chem. Phys. 1999, 111, 572–582.

[30] T. Ebata , M. Hamakado , S. Moriyama , Y. Morioka , M. Ito , Chem. Phys. Lett. 1992, 199, 33–41.

[31] S. Tsuzuki , K. Honda , T. Uchimaru , M. Mikami , J. Chem. Phys. 2005, 122, 144323. 15847538 10.1063/1.1876092

[32] P. Hobza , V. Spirko , H. L. Selzle , E. W. Schlag , J. Phys. Chem. A 1998, 102, 2501–2504.

[33] P. Hobza , H. L. Selzle , E. W. Schlag , J. Phys. Chem. 1996, 100, 18790–18794.

[34] M. O. Sinnokrot , C. D. Sherrill , J. Phys. Chem. A 2006, 110, 10656–10668. 16970354 10.1021/jp0610416

[35] C. D. Sherrill , T. Takatani , G. H. Hohenstein , J. Phys. Chem. A 2009, 113, 10146–10159. 19689152 10.1021/jp9034375

[36] F. L. Gervasio , R. Chelli , P. Procacci , V. Schettino , J. Phys. Chem. A 2002, 106, 2945–2948.

[37] M. O. Sinnokrot , C. D. Sherrill , J. Am. Chem. Soc. 2004, 126, 7690–7697. 15198617 10.1021/ja049434a

[38] C. M. Baker , G. H. Grant , Biopolymers 2007, 85, 456–470. 17219397 10.1002/bip.20682

[39] W. L. Jorgensen , E. R. Laird , T. B. Nguyen , J. Tirado-Rives , J. Comput. Chem. 1993, 14, 206–215.

[40] W. L. Jorgensen , D. S. Maxwell , J. Tirado-Rives , J. Am. Chem. Soc. 1996, 118, 11225–11236.

[41] M. I. Cabaço , Y. Danten , M. Besnard , Y. Guissani , B. Guillot , J. Phys. Chem. B 1997, 101, 6977–6987.

[42] R. Righini , Science 1993, 262, 1386. 17736818 10.1126/science.262.5138.1386

[43] P. M. Zorkii , L. V. Lanshina , T. V. Bogdan , J. Struct. Chem. 2008, 49, 524–527.

[44] C. Chipot , R. Jaffe , B. Maigret , D. A. Pearlman , P. A. Kollman , J. Am. Chem. Soc. 1996, 118, 11217–11224.

[45] C. M. Baker , G. H. Grant , J. Chem. Theory Comput. 2006, 2, 947–955. 26633054 10.1021/ct060024h

[46] M. Fioroni , D. Vogt , J. Phys. Chem. B 2004, 108, 11774–11781.

[47] T. F. Headen , C. A. Howard , N. T. Skipper , M. A. Wilkinson , D. T Bowron , A. K. Soper , J. Am. Chem. Soc. 2010, 132, 5735–5742. 20102204 10.1021/ja909084e

[48] M. Falkowska , D. T. Bowron , H. G. Manyar , C. Hardacre , T. G. Youngs , ChemPhysChem 2016, 17, 2043–2055. 26990367 10.1002/cphc.201600149 PMC4999024

[49] G. N. Patwari , P. Vanuvanalingam , M. Kołaski , Chem. Phys. 2013, 415, 150–155.

[50] S. Maity , G. N. Patwari , R. Sedlak , P. Hobza , Phys. Chem. Chem. Phys. 2011, 13, 16706–16712. 21858279 10.1039/c1cp20677j

[51] A. Kundu , S. Sen , G. N. Patwari , Phys. Chem. Chem. Phys. 2015, 17, 9090–9097. 25758455 10.1039/c5cp00162e

[52] C. A. Hunter , J. K. Sanders , J. Am. Chem. Soc. 1990, 112, 5525–5534.

[53] C. A. Hunter , Chem. Soc. Rev. 1994, 23, 101–109.

[54] M. O. Sinnokrot , C. D. Sherrill , J. Phys. Chem. A 2003, 107, 8377–8379.

[55] S. E. Wheeler , K. N. Houk , J. Am. Chem. Soc. 2008, 130, 10854–10855. 18652453 10.1021/ja802849j PMC2655233

[56] F. Cozzi , R. Annunziata , M. Benaglia , K. K. Baldridge , G. Aguirre , J. Estrada , Y. Sritanna-Anant , J. S. Siegel , Phys. Chem. Chem. Phys. 2008, 10, 2686–2694. 18464983 10.1039/b800031j

[57] F. Cozzi , M. Cinquini , R. Annunziata , T. Dwyer , J. S. Siegel , J. Am. Chem. Soc. 1992, 114, 5729–5733.

[58] C. J. Benmore, A. K. Soper, *The SANDALS Manual: a Guide to Performing Experiments on the Small Angle Neutron Diffractometer for Amorphous and Liquid Samples at ISIS. Version 1.0*, RAL Technical Reports, RAL-TR-1998–006, **1998**.

[59] A. K. Soper, *GudrunN and GudrunX: programs for correcting raw neutron and X-ray diffraction data to differential scattering cross section*, Science & Technology Facilities Council **2011**.

[60] A. K. Soper, A. K. *Empirical potential structure refinement-EPSRshell: a user's guide*, In Version 18, Technical Report RAL-TR-2011–012, **2011**.

[61] W. Jorgensen , J. Tirado-Rives , J. Am. Chem. Soc. 1988, 110, 1657–1666. 27557051 10.1021/ja00214a001

[62] T. G. Youngs, dlputils, https://www.projectaten.com/dlputils, v. 1.4.3.

