# Supplementary material for: The Structure of Ethylbenzene, Styrene and Phenylacetylene Determined by Total Neutron Scattering
Source: Chemphyschem. 2017 Aug 16;18(18):2541–8. doi: 10.1002/cphc.201700393 (PMC5811833; doi:10.1002/cphc.201700393)
Supplement: Supplementary file 1 — Supplementary [file CPHC-18-2541-s001.pdf]

CHEMPHYSCHEM

Supporting Information

The Structure of Ethylbenzene, Styrene and Phenylacetylene Determined by Total Neutron Scattering

Joanna Szala-Bilnik,[a, b] Marta Falkowska,[b, c] Daniel T. Bowron,[b] Christopher Hardacre,*[a] and Tristan G. A. Youngs*[b]

cphc_201700393_sm_miscellaneous_information.pdf

Supporting Information

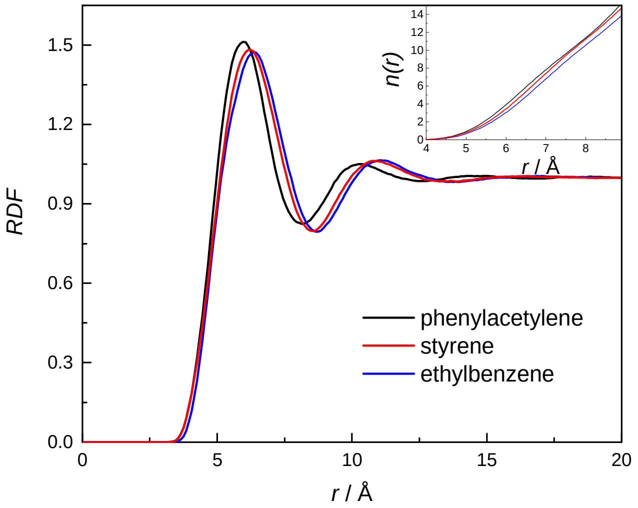

Figure S1. Molecular centre of geometry radial distribution functions for phenylacetylene (black), styrene (red) and ethylbenzene (blue). Insert: coordination number.

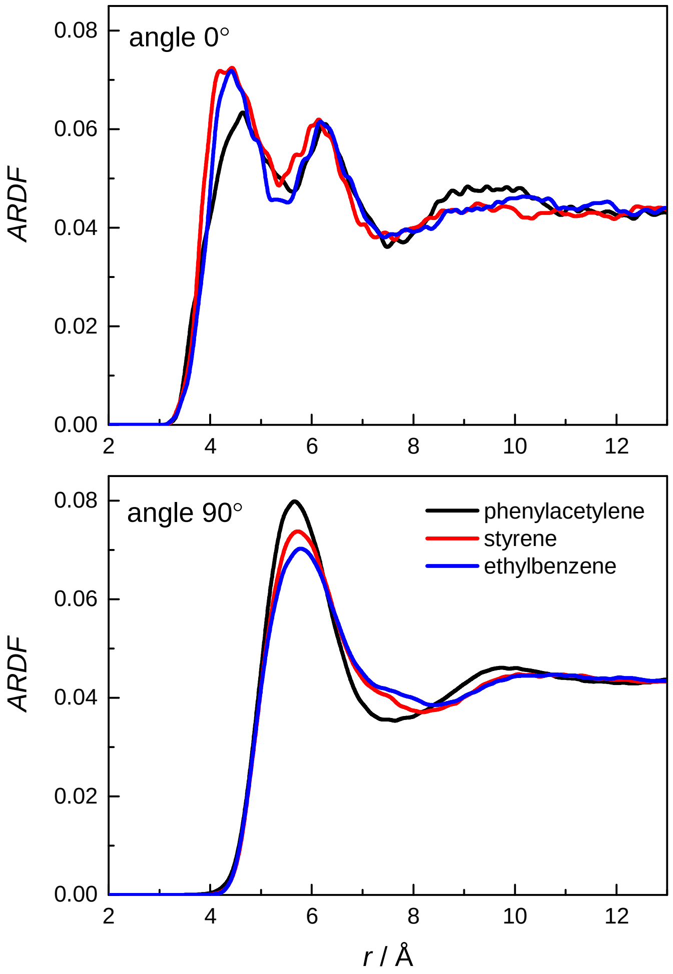

Figure S2. Angular radial distribution function for phenylacetylene, styrene and ethylbenzene calculated as a function of the angle (shown in Figure 3) between the *z* axes of the central and surrounding molecules. The angle between the two molecules are 0° (top) and 90° (bottom). The black lines correspond to phenylacetylene, red lines to styrene and blue lines to ethylbenzene.

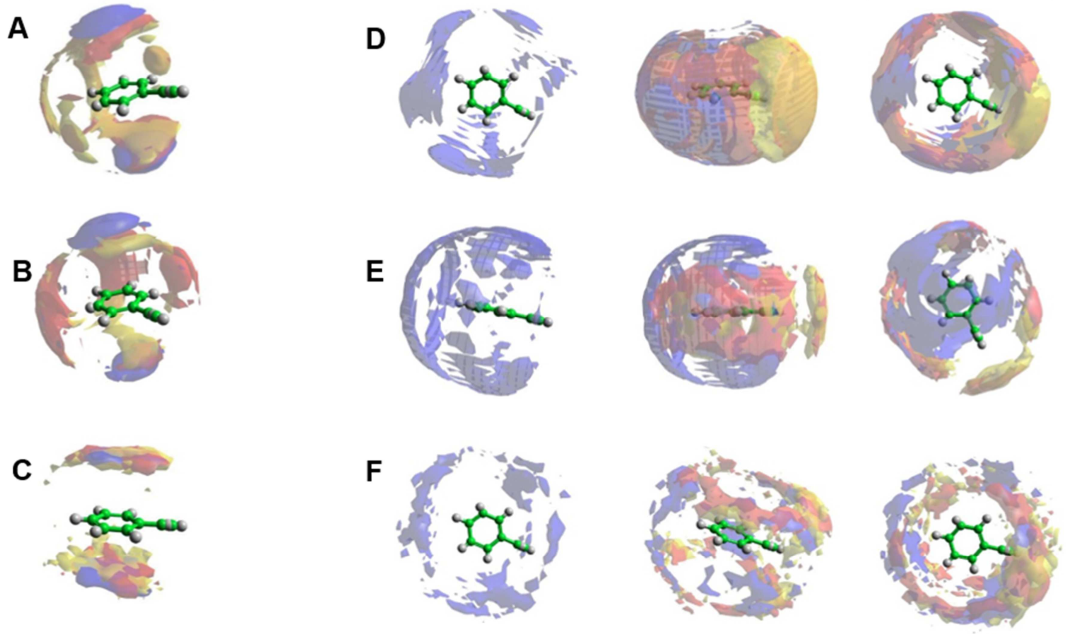

Figure S3. Spatial probability densities for liquid styrene calculated within two distances ranges determinate from ARDF, that is, 0 – 5.35 Å and 5.35 – 7.65 Å from the central molecule. The function represents the top 20 % of (A) all molecules, (B) perpendicular molecules only (θ = 90 ± 10°) and (C) parallel molecules only (θ = 0 ± 10°) with respect to the central molecule found within r = 0 – 5.35 Å. Additionally, functions representing the top 10% of (D) all molecules, (E) perpendicular molecules only (θ = 90 ± 10°) and (F) parallel molecules only (θ = 0 ± 10°) with respect to the central molecule found within r = 5.35 – 7.65 Å. Three different perspectives with only centre of geometry (blue) and centre of geometry and C11 (red) and C13 atoms (yellow surface) are shows for D, E and F to illustrate the structures.

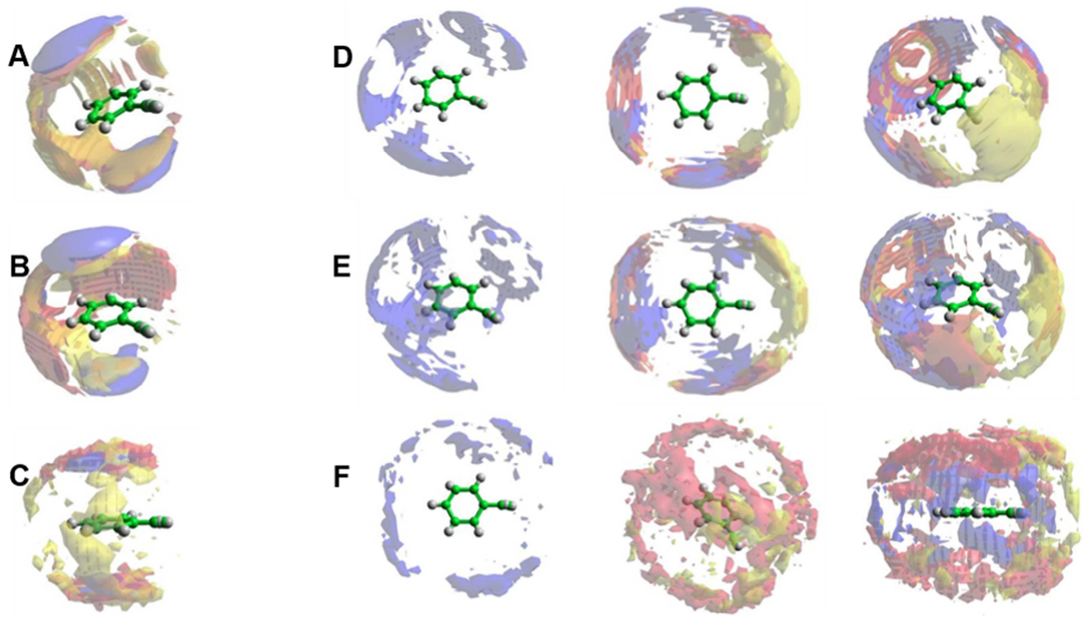

Figure S4. Spatial probability densities for liquid ethylbenzene calculated within two distances ranges determinate from ARDF, that is, 0 – 5.55 Å and 5.55 – 7.45 Å from the central molecule. The function represents the top 20 % of (A) all molecules, (B) perpendicular molecules only (θ = 90 ± 10°) and (C) parallel molecules only (θ = 0 ± 10°) with respect to the central molecule found within r = 0 – 5.55 Å. Additionally, functions representing the top 10% of (D) all molecules, (E) perpendicular molecules only (θ = 90 ± 10°) and (F) parallel molecules only (θ = 0 ± 10°) with respect to the central molecule found within r = 5.55 – 7.45 Å. Three different perspectives with only centre of geometry (blue) and centre of geometry and C11 (red) and C13 atoms (yellow surface) are shows for D, E and F to illustrate the structures.

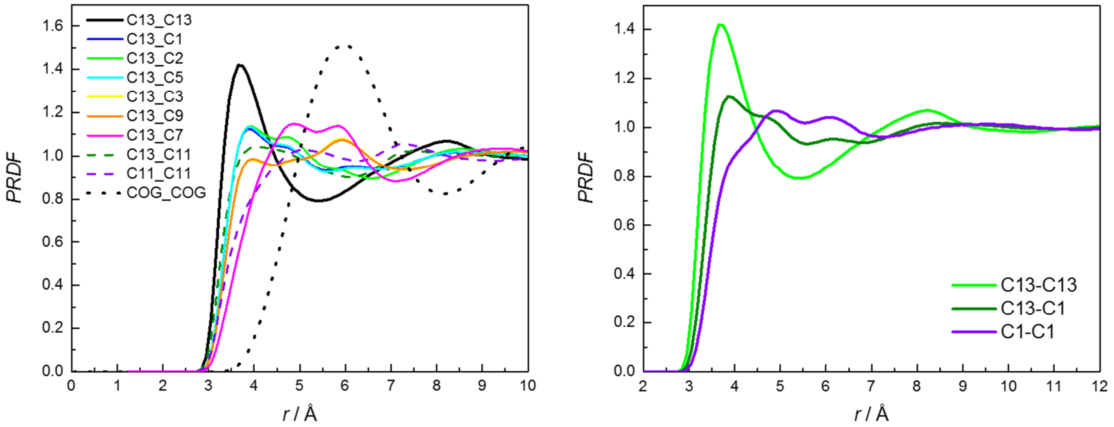

Figure S5. Site–site radial distribution functions for phenylacetylene.

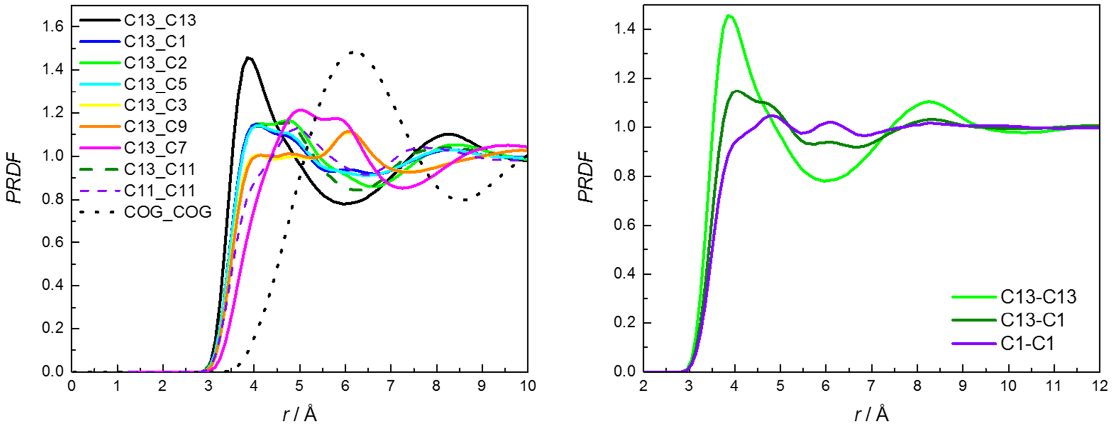

Figure S6. Site–site radial distribution functions for styrene.

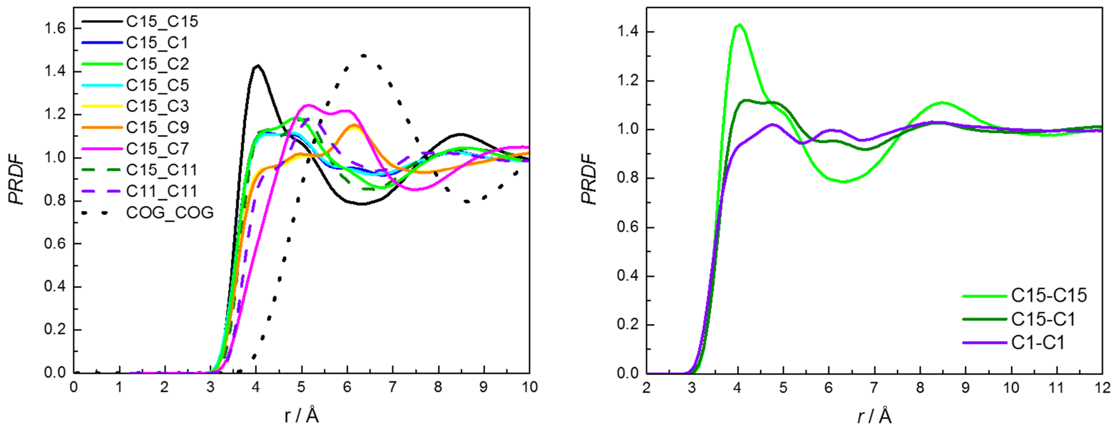

Figure S7. Site–site radial distribution functions for ethylbenzene.

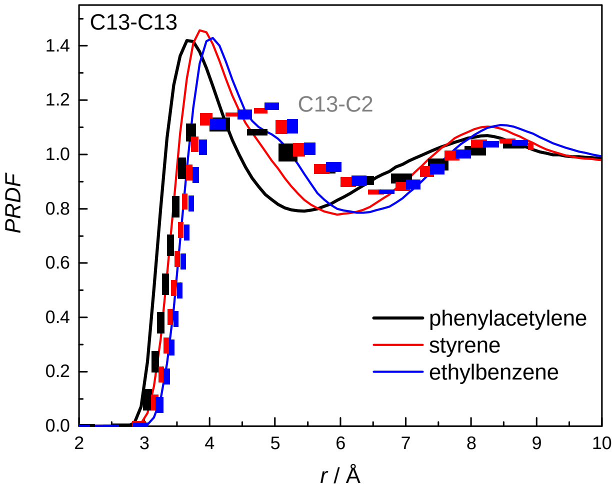

Figure S8. Molecular site–site radial distribution functions of C13 – C13 (solid line) and C13 – C2 (dash line) for phenylacetylene (black), styrene (red) and ethylbenzene (blue). Inset: C13 – C2 for three solvents.
